# The skeleton: an overlooked regulator of systemic glucose metabolism in cancer?

**DOI:** 10.3389/fonc.2024.1481241

**Published:** 2024-11-11

**Authors:** Rucha Ronghe, Adriana A. S. Tavares

**Affiliations:** ^1^ Edinburgh Medical School, The University of Edinburgh, Edinburgh, United Kingdom; ^2^ University/British Heart Foundation Centre for Cardiovascular Science, The University of Edinburgh, Queens Medical Research Institute, Edinburgh, United Kingdom; ^3^ Edinburgh Imaging, The University of Edinburgh, Queens Medical Research Institute, Edinburgh, United Kingdom

**Keywords:** glucose, bone, metabolism, cancer, systemic

## Abstract

Recent discoveries demonstrated the skeleton’s role as an endocrine organ regulating whole-body glucose homeostasis. Glucose metabolism is critical for rapid cell proliferation and tumour growth through increasing glucose uptake and fermentation of glucose to lactate despite being in an aerobic environment. This hypothesis paper discusses emerging evidence on how bones can regulate whole-body glucose homeostasis with potential to impact on tumour growth and proliferation. Moreover, it proposes a clinical link between bone glucose metabolism and prognosis of cancer based on recent clinical trial data. Targeting metabolic pathways related with classic glucose metabolism and also bone metabolism, novel methods of cancer therapy and treatment could be developed. This paper objective is to highlight the need for future research on this altered metabolism with potential to change future management of cancer patients.

## Introduction

Historically it was believed that human diseases were compartmentalised to different organs. However, the human body is a multi-dimensional network of interconnected organs and diseases that materialise at many levels of complexity frequently including multiple systems (1, 2). This review aims to propose and discuss a potential link between the skeletal system and tumour growth and proliferation through glucose affecting both cancer and whole-body metabolism.

Bones have classically been considered as rigid structures, providing support and protection to organs (3). The skeleton undergoes continuous lifelong remodelling to sustain its changing mechanical needs and repair micro-damages (4). This is achieved by specialised cells, osteoblasts, osteoclasts and osteocytes which perform specific roles. Recent discoveries in bone biology have identified the skeleton not only as a structural supportive network but also as an endocrine organ itself. These advances reveal that bones act as key regulators of whole-body glucose metabolism and elucidate the many novel pathways and mechanisms by which bones achieve this (5). Given these recent results showing that bone-derived hormones are involved in regulation of whole-body glucose homeostasis, it could be hypothesised that bone health could impact systemic glucose homeostasis potentially accelerating development and progression of systemic disease such as cancer.

Cancer is one of the leading causes of death worldwide (6). In the US alone, 1,958, 310 cases of cancer were diagnosed in 2023 (7) The American Cancer society estimates that 400,000 new cases of malignant bone metastasis are diagnosed in the United States each year (8). Therefore, its gravity cannot be overlooked. Cancer is a process that starts locally but can develop to have systemic effects and it is common for bones to be sites of metastases. The cause for cancer related mortality to be high is due to metastases which occur due to dissemination of tumour cells from the primary site via circulation or lymphatic system (9). It is known that only a small fraction of disseminating tumour cells cause cancer metastases however if the primary tumour spreads to bone, osteolytic or osteoblastic metastases can occur resulting in a vicious cycle of bone destruction and tumour growth (10, 11). Osteolytic bone metastases are more common in breast, lung, renal, thyroid cancers and in multiple myeloma. Osteoblastic bone metastases are most prevalent in prostate cancers but also exist in breast, colon and cervical cancers (12). Bone is the most common site of secondary metastases, following lung and liver. Metastatic cells from primary tumours travel through the lymphatic system to bone, where they continue to grow the tumour. Bone metastases can occur in almost all tumours, but breast and prostate cancer are the most common to develop in skeletal metastases (13). The relative incidence of bone metastasis in patients with advanced metastatic disease is as follows; 65-75% in breast cancer; 65-75% in prostate; 60% in thyroid; 30-40% in lung; 40% in bladder; 20-25% in renal cell carcinoma (14).

**Figure 1:**
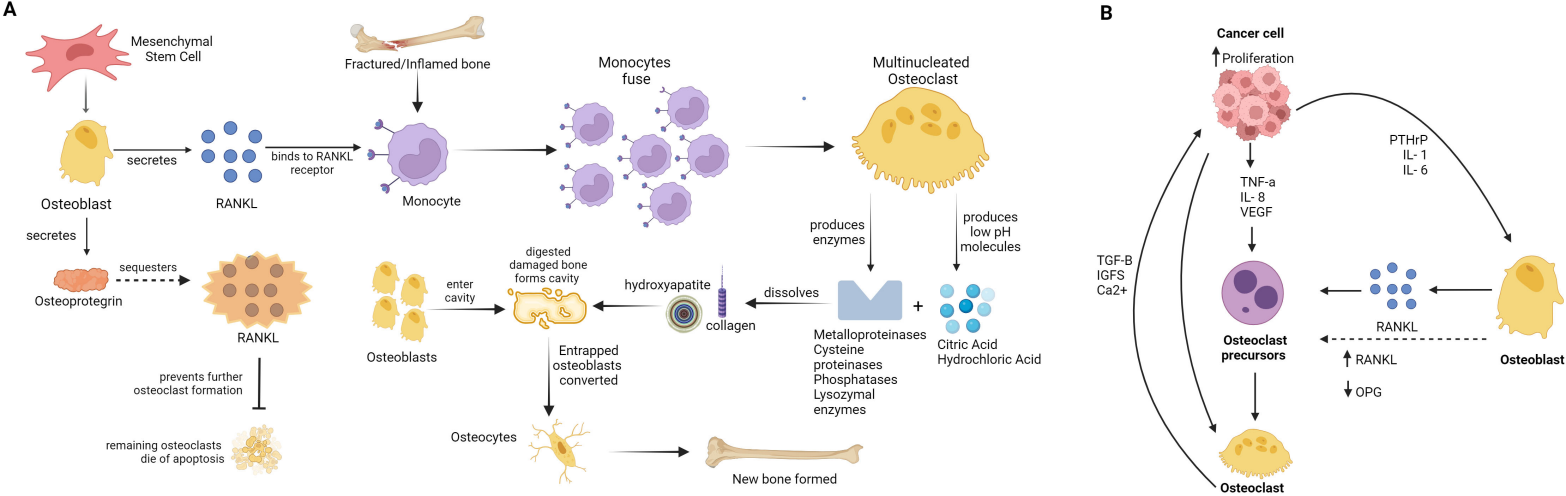
Scheme of bone remodelling and cancer proliferation and metastisation. **(A)** Bone remodelling cycle: The diagram shows the normal process of bone remodelling, where osteoclasts and osteoblasts coordinate to maintain bone health. Osteoblasts originate from mesenchymal stem cell (MSC) producing RANKL, RANKL binds to RANKL-receptor on monocytes in fractured or injured/inflamed bone. Monocytes fuse to form multi-nucleated osteoclast, osteoclast produces enzymes like metalloproteinases (MMPs), cysteine proteinases (including cathepsin K), phosphatases, lysozymal enzymes, and low pH molecules including citric acid and hydrochloric acid, that digest damaged bone area (dissolving collagen and hydroxyapatite) forming cavity. Osteoblast produced osteoprotegrin, which sequesters RANKL and prevents further osteoclast formation, while previously formed osteoclast die. Osteoblast enter cavity formed by osteoclast activity and form new bone. Entraped osteoblast in new bone-mass, converts to osteocyte. **(B)** The development of osteoclastic and osteoblastic bone metastases: The formation of osteoclastic and osteoblastic bone metastases involves interactions between tumour cells and bone cells in the bone microenvironment. Tumour cells release factors that enhance the activity of osteoclasts (osteoclastogenesis) and osteoblasts (osteoblastogenesis). As osteoclasts mature, they release factors such as Insulin-like growth factor 1 (IGF-1) and transforming growth factor beta (TGF-β), which help tumour cells survive and multiply, further promoting the cycle of bone resorption and formation. This interaction fosters a conducive environment for tumour growth within the bone.

Bone homeostasis is controlled by a tightly regulated balance between bone deposition and bone resorption. However cancers that metastasise to bone or cancer treatments such as chemoradiation can severely disrupt bone homeostasis (15). In bone metastasis, cancer cells stimulate osteoclasts, which are responsible for bone resorption, leading to excessive bone degradation (**Figure 1**). At the same time, osteoblast activity, which is essential for depositing new bone, is suppressed or insufficient, resulting in a net loss of bone mass and structural integrity (16). This imbalance between bone resorption and formation can lead to skeletal complications. When bones are metastasised by cancer cells such those from breast cancer, osteoblasts increase their production of inflammatory cytokines including interleukin-6 (IL-6), monocyte chemoattractant protein-1 (MCP-1), regulated on activation normal T cell expressed and secreted (RANTES), and macrophage inflammatory protein-1 alpha (MIP-1 alpha); factors that stimulate osteoclastogenesis including granulocyte-colony stimulating factor (G-CSF), RANK-L, and granulocyte macrophage-colony stimulating factor (GM-CSF); and cytokines that recruit both innate and adaptive immune cells including interleukin-8 (IL-8), interleukin-12 (IL-12), and interferon gamma-induced protein 10 (IP-10) (17, 18).

Unfortunately, bone metastases can often cause limb dysfunction, pathological fractures, spinal cord compression and severe pain, and is associated with significant morbidity, reduction in quality of life and poor prognoses (19).

## Glucose and cancer initiation, progression and treatment

The metabolism of glucose allows for energy to be utilised in the form of ATP through oxidation of its carbon bonds. This process is essential for life. The end products of this reaction in cases of full oxidation of glucose through respiration in the mitochondria are CO_2_, or lactate in hypoxic conditions (20). In 1924, Warburg described a phenomenon in which cancer cells opt for glycolysis as their primary energy source even in aerobic conditions (21). Evidence confirms that tumour and proliferative cells metabolise up to ten-fold more lactate when compared to normal cells under aerobic conditions. This process is known as the Warburg effect (22).

**Figure 2:**
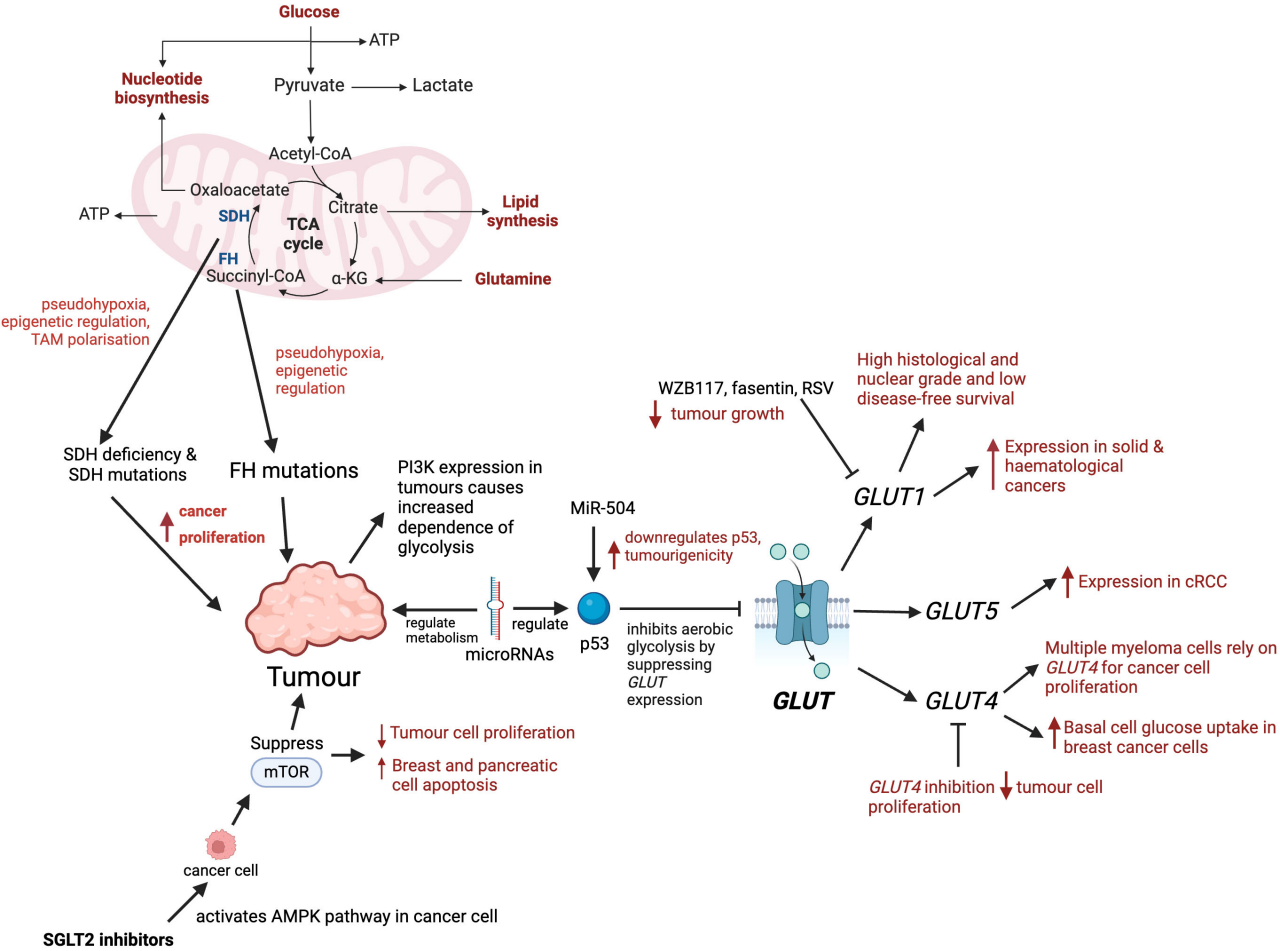
Role of glucose metabolism and transporters in tumour progression. Mutations in key enzymes such as SDH and FH disrupt the TCA cycle causing pseudohypoxia, epigenetic changes, and tumour-associated macrophage (TAM) polarisation, which promote tumour proliferation. PI3K expression in tumours increases their dependence on glycolysis. microRNAs regulate glucose metabolism by targeting enzymes, transcription factors, oncogenes or tumour suppressors. microRNA-504 downregulates p53, enhancing tumour growth. microRNAs regulate p53, and p53 inhibits aerobic glycolysis by supressing *GLUT* expression. *GLUT1* is expressed in solid and haematological cancers, and its overexpression is associated with high histological grade and poor survival. Inhibitors of *GLUT1*, such as fasentin, WZB117 and RSV have been shown to reduce tumour progression. Multiple myeloma cells rely on *GLUT4* for glucose consumption allowing greater cancer cell proliferation. *GLUT4* has been identified to have an important role in basal glucose uptake in breast cancer cells. *GLUT4* inhibition reduces tumour cell proliferation and affects cell survival under hypoxic conditions. cRCC’s have high glycolytic rates and shown to express *GLUT5.* SGLT2 inhibitors initiate AMPK pathway in cancer cells which results in the inhibition of mTOR, and in turn causing apoptosis of cancer cells.

Sugar cannot diffuse across the phospholipid bilayer, hence glucose can be considered as the rate-limiting step for tumour progression (23) (**Figure 2**). It is important to study these metabolic pathways since cell signalling pathways are important in cancer cell growth and proliferation. Regulation of these pathways allows the amalgamation of nutrients essential for tumour cells into biomass (24). Moreover, it has been identified that activation of certain oncogenes including *c-myc*, *ras* and *src*, as well as transcription factors such as hypoxia inducible factor-1*a* can result in overexpression and activity of glycolytic enzymes and GLUTs (25). Moreover, these oncogenes are known to regulate metabolic phenotypes of tumours and play a significant role in how the tricarboxylic acid cycle (TCA cycle) is utilised in these cancer cells (26).

The TCA cycle is a fundamental metabolic pathway that allows cells to utilise glucose, amino and fatty acids (27). Despite the evidence that cancer cells do not take part in the TCA cycle, upcoming research highlights that various cancer cells, in particular those with deregulated oncogene and tumour gene suppression actually heavily rely on TCA as an energy source (28). Moreover, in many types of cancer there are mutations that affect the integrity of this cycle, in turn the dysregulation in the production of the TCA cycle metabolites (29). The biochemical reactions in the TCA cycle are catalysed by multiple enzymes and recent research demonstrates these enzymes can undergo mutations or can be dysregulated in a spectrum of cancer types. Succinate dehydrogenase (SDH) has roles in the TCA cycle and mutations in this enzyme have been identified in gastrointestinal stromal tumours, renal tumours, thyroid tumours and many more implicating the significance of SDH in a variety of cancers (30). Furthermore, SDH deficiency has been shown to promote cancer proliferation and metastasis by inducing pseudohypoxia, epigenetic regulation, and tumour assisted macrophages (TAM) polarisation (31). Similarly, heterogeneous mutations of fumarate hydratase (FH) in the TCA cycle, predispose individuals to multiple cutaneous and uterine leiomyomas, hereditary leiomyomatosis and renal cell cancer (32). Furthermore, mutations in FH have been identified in bladder, breast and testicular cancer (33). A study has shown that FH promotes cancer proliferation and metastasis by inducing pseudohypoxia, epigenetic regulation of gene expression (34). These enzymes are an integral part of the TCA cycle and it could be hypothesised that they have the potential to affect cancer progression. A further understanding of TCA metabolites roles and molecular mechanisms in oncogenesis is required which could prompt novel metabolite-based cancer therapy.

The cellular transport of sugars requires transport proteins to mediate uptake or release from cells. Currently there are three families of glucose transporters that have been identified, including sodium-driven glucose symporters (SGLTs), glucose transporters (GLUTs) and more recently SWEETs, which have not yet been explored in mammals, but in plants they are mainly responsible for efflux and intracellular trafficking of sugars (35). These glucose transporter families have distinct mechanisms and physiological functions. SGLT carriers are important for glucose absorption in the small intestine and glucose reabsorption in the renal cortex. They facilitate the translocation of glucose against its own concentration gradient using the energy released from the downhill flow of Na^+^ (36). With regards to the GLUT family there are 14 members that have been identified in humans which can be subdivided into three classes. *GLUTs* catalyse the diffusion of glucose along its own concentration gradient (37).

*GLUT1* is expressed in erythrocytes, placenta and endothelial cells and regulates entry across the blood brain barrier (38). In comparison with other *GLUTs*, *GLUT1* has a high selectivity for glucose thus playing an imperative role in those tissues that rely on glucose for their energy source (39). *GLUT1* expression is an important hallmark for many cancers and studies demonstrate that *GLUT1* has been overexpressed in both solid and haematological malignancies (**Figure 2**). Some of these cancer types where *GLUT1* has been found to be overexpressed include breast cancer, non-small cell lung cancer, large B-cell lymphoma, head and neck cancers and glioblastomas (40). Moreover, another study reinforced this by comparing the length time of disease-free survival in patients with *GLUT1* expression in breast cancer cells and those without, and it was much shorter in patients with *GLUT1* expression compared to those without. *GLUT1* expression was associated with pathological poor prognostic factors such as high histological and nuclear grade and low disease-free survival. This suggests that *GLUT1* could be used as a prognostic marker in breast cancer patients and could also potentially be used as a target in personalised treatment approaches (41).

Additionally, in breast cancer, non-small cell lung cancer, large B-cell lymphoma, head and neck cancers and glioblastomas cancer types there is data that supports the concept that *GLUT1* overexpression correlates with the grade and stage of tumours and the patients clinical outcome (42). Studies have shown that clear renal cell carcinomas (cRCC) have high glycolytic rates and express *GLUT* transporters (43). The Lidgren et al. study had 80 samples of cRCCs and through creating an isoform of *GLUT5*, many cancer pathological parameters such as grade of differentiation, pelvis invasion and breaking capsule were shown to have a relationship with *GLUT5*. The expression of *GLUT5* correlated more with cRCC in comparison to renal cell carcinoma (44). This data further suggests that the isoform of *GLUT5* in fructose uptake in cRCC could potentially have a therapeutic benefit to hinder the progression to malignant renal cell carcinoma (45) (**Figure 2**).

*GLUT 4* is primarily found in adipose tissues and striated muscle (skeletal and cardiac) (46). Studies have shown that multiple myeloma cells rely on *GLUT4* for glucose consumption allowing greater cancer cell proliferation, viability and survival (47). Moreover, *GLUT4* has been identified to have an important role in basal glucose uptake in MCF7 and MDA-MB-231 breast cancer cells (48). When *GLUT4* is downregulated, there is a diminished glucose uptake and forced metabolic reprogramming into oxidative phosphorylation. This reallocation causes an increase in activity of aerobic respiration, reducing the production of lactate. As a result *GLUT4* inhibition reduces tumour cell proliferation and affects cell survival under hypoxic conditions (49). Additionally, the majority of gastrointestinal stromal tumours have a gain of function mutation called c-KIT. It has been shown that gastrointestinal stromal tumour cells after treatment with imatinib, have reduced the expression of *GLUT4* in the plasma membrane (50). This is because imatinib, which reduces glucose uptake via decreased levels of plasma membrane-bound *GLUT4*, induces apoptosis or growth arrest by inhibiting c-KIT activity (50).

Interestingly, several inhibitors of glucose transporters, such as fasentin, phloretin, STF-31, and WZB117 have already been discovered, and experiments with preclinical models demonstrated their impact in reducing tumour progression (51–53). WZB117 is a small molecule which inhibits GLUT1 and it acts on cancer cells *in vitro* and *in vivo* (54). Inhibition of GLUT1 by WZB117 decreases the levels of intracellular ATP and glycolytic enzymes. Animal studies performed on mice revealed that WZB117 inhibits cancer growth. After daily intraperitoneal injection of this inhibitor, the sizes of the compound-treated tumours were on average more than 70% smaller in comparison with control animals (55). Moreover, fasentin, an inhibitor of GLUT1, which binds directly to GLUT1 and inhibits glucose uptake, increases apoptosis in prostate cancer, multiple myeloma cells, and acute promyelocytic leukemia cells. It sensitises these cancer cells to FAS ligand-death receptor signalling (51). Moreover, Resveratol (RSV), is a polyphenolic natural product that attracted great interest mainly due to its anticarcinogenic, anti-inflammatory, and cardioprotective properties (56). RSV has structural similarities with tyrosine kinases that are known inhibitors of GLUT1 (57). In cancer cells it is observed that resveratrol inhibited the uptake of glucose, favouring the anticancer action. The type of cell death observed in ovarian cancer cells treated with RSV has been reported as apoptosis or autophagy (58). Thus these inhibitors of GLUT transporters are prototypes for further development of anticancer therapeutics targeting GLUT mediated glucose transport and glucose metabolism (**Figure 2**).

SGLT-2 inhibitors are a new class of antidiabetic drugs which act by decreasing the active reverse transport of glucose by SGLT-2 in renal tubule. SGLT-2 inhibitors reduce glucose absorption, induce glycosuria which lowers plasma glucose independent of insulin. Preclinical and *in vitro* cell studies have confirmed that SGLT-2 inhibitors have anti-proliferative effects of certain cancers including liver, pancreatic, bowel, lung and breast cancers (59). One proposed mechanism of action of SGLT-2 inhibitors is the inhibitory effect of the production of ATP, which causes the activation of the AMPK pathway in cancer cells. In turn, this SGLT-2 inhibitor induced AMPK activation results in inhibition of mTOR (60). mTOR signalling is important in cell growth and metabolism and is dysregulated in cancer pathophysiology (61). This inhibition of mTOR results in initiation of apoptosis (62).

The metabolism, proliferation, growth and survival of cancer cells is also largely dependent on signalling through the serine/threonine-protein kinase (AKT) and phosphoinositide-3-kinase (PI3K) pathways (63). The cells in the AKT pathway are those which are most frequently activated and proliferate and survive in the cancer pathway. Agents that target PI3K are being researched in clinical trials and there is upcoming evidence that expression of PI3K causes an increased dependence of glycolysis in turn implying that drugs targeting the PI3K pathway would interfere with glucose metabolism (24).

Finally, there has been evidence to suggest that microRNAs regulate cancer metabolism that evince a higher rate of metabolism of glucose. MicroRNAs are a family of non-coding RNAs that are not translated into proteins but control gene expression either at pre- or post-transcriptional stages. Moreover, they have been shown to be involved in multiple biological processes, one of which is glucose metabolism, mediated by targeting enzymes, transcription factors, oncogenes or tumour suppressors (64). P53 is a tumour suppressor gene, meaning that it functions to inhibit the growth of tumours, cell cycle progression, apoptosis and DNA damage response (65). A number of microRNAs regulate p53. The mechanism of p53 is that it inhibits aerobic glycolysis by suppressing expression of some *GLUTs* and other molecules. As a result, the dysregulation of p53 that occurs in many malignancies leads to pentose phosphate pathway or aerobic glycolysis (66). MiR-504 has been shown to downregulate p53 and increases tumourigenicity (67).

To sum up, previous studies have confirmed that glucose does play a critical role in the cancer metabolic pathways through various mechanisms described above. A better understanding of how cancer cells rely on systemic glucose metabolism for initiation and progression will hopefully lead to novel targeting modalities for cancer treatment. As discussed, there is new research into how bones regulate whole-body glucose metabolism therefore there could be a potential link between the skeletal system affecting cancers. This will be discussed in further detail in the next section of this hypothesis paper.

## Role of bones in regulating systemic glucose metabolism: key mechanisms and mediators

The human adult skeleton consists of 213 bones, not including the sesamoid bones. Bones vary in size, shape and strength to respond to the demands of the motor tasks.

Bones are made up of osteoid matrix and hydroxyapatite [Ca_10_(PO_4_)_6_(OH)_2_] crystal but they also contain water, non-collagenous and collagenous proteins, lipids and specialised bone cells (68). Bone mineral, in the form of hydroxyapatite crystals, is an essential store of calcium and phosphate crucial for mineral homeostasis and provides the skeleton with mechanical rigidity and compressive strength.

The musculoskeletal system constantly undergoes remodelling to replace old and damaged bone to maintain bone integrity and mineral homeostasis (69). The bone remodelling cycle consists of five key co-ordinated steps; activation, resorption, reversal, formation and termination which takes approximately 120 to 200 days in cortical and trabecular bone, respectively (70). Osteoclasts are cells that break down and resorb bone, and osteoblasts are cells that form new bone tissue (71). During the bone remodelling cycle, osteoclastic resorption is tightly coupled with osteoblastic bone formation (72). The remodelling cycle has two key pathways receptor activator of NF-kB (RANK)/receptor activator of NF-kB ligand (RANKL)/osteoprotegerin (OPG) and wingless-related integration site (Wnt) that transduce systemically and locally produced signals (73). These pathways are important in determining the timing and balance of bone resorption and formation to ensure its tight regulation (74). Bone remodelling also plays an important role in maintaining plasma calcium homeostasis (75). The regulation of bone remodelling is both systemic and local. The major systemic regulators include parathyroid hormone (PTH), calcitriol, growth hormone, glucocorticoids, thyroid hormones, and sex hormones. Others such as insulin-like growth factors (IGFs), prostaglandins, tumour growth factor-beta (TGF-beta), bone morphogenetic proteins (BMP), and cytokines are also crucial (76). This cycle is a life-long process and is imperative for preserving bone integrity and strength.

**Figure 3:**
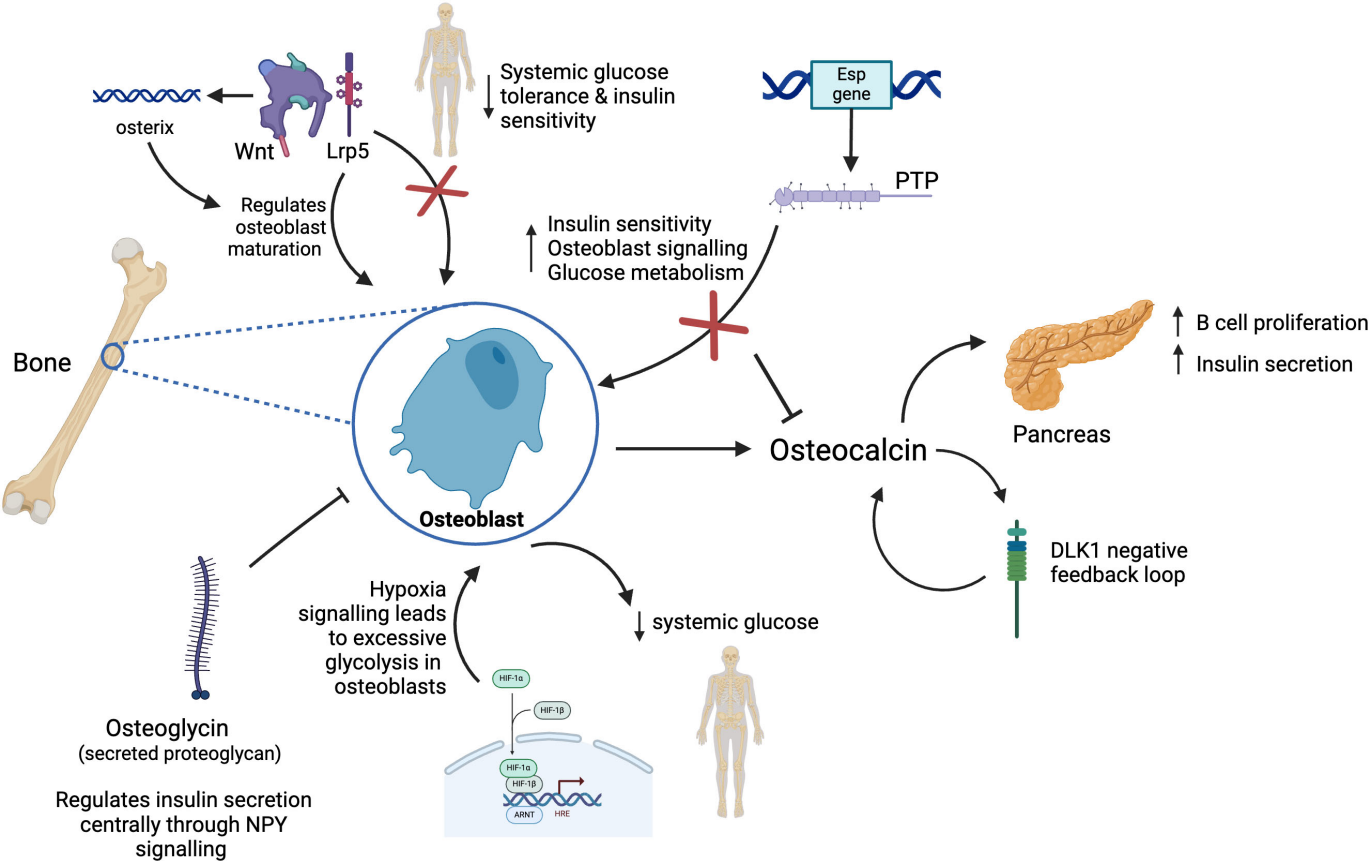
Model of new endocrine functions of bone. Osteoblasts in bone release the hormone osteocalcin which is responsible for multiple endocrine functions including acting on the pancreas to increase insulin secretion and beta cell proliferation. Osteocalcin controls the production of DKL1 which works as a negative feedback system preventing osteocalcin induced hypoglycaemia. Osteoblasts express *Esp* gene which codes for PTP, in turn this controls glucose metabolism by *Esp* negatively regulating osteocalcin resulting in reduced bone resorption and insulin secretion. Osteoglycin inhibits osteoblast activity and regulates glucose homeostasis through NPY. Wnt signalling through Lrp5 signalling regulates osteoblast maturation and whole-body glucose metabolism. Activation of Wnt pathway stimulates production of osterix, a transcription factor further promoting osteoblast differentiation. Hypoxia signalling through HIF-1 leads to excessive glycolysis in osteoblasts, thus reducing systemic levels of glucose.

Osteoblasts are known to release the hormone osteocalcin, promoting whole-body glucose homeostasis (**Figure 3**). Moreover, uncarboxylated osteocalcin has less affinity for hydroxyapatite, readily promoting its release into circulation, and it is responsible for multiple endocrine functions (77). A pre-clinical study demonstrated that mice lacking in osteocalcin were obese, hyperglycaemic, hypoinsulinemic with reduced insulin secretion and sensitivity. They had reduced islet size and beta cell mass, suggesting a link between bone and regulation of energy metabolism (78). Conversely, one study using osteocalcin deficient mice showed no impact on glucose metabolism and muscle mass in mice (79). Notwithstanding, osteoblasts express the *Esp* gene, coding for protein tyrosine phosphatase (PTP), which controls glucose metabolism by *Esp* negatively regulating osteocalcin by producing its inactive form. Pancreas-specific PTP depleted mice demonstrate impaired glucose tolerance and glucose-stimulated insulin secretion when tested with high fat feeding (**Table 1**). Mice lacking PTP in osteoblasts demonstrated an increase in bone resorption and insulin sensitivity (80). Pre-clinical studies highlighted that mice without *Esp* had increased uncarboxylated osteocalcin resulting in increased osteoblast signalling and whole-body glucose metabolism (81, 82). However this is contradicted by a study (83) suggesting that mice without *Esp*, appeared hypoglycaemic and severely sensitive to insulin. Interestingly, osteoblasts express copious amounts of insulin receptor and respond to insulin by upregulating the expression of anabolic bone markers, increasing glucose uptake along with several other functions (81). There has been evidence to show that localised insulin quickens bone fracture healing and formation *in vivo* in comparison to patients with Type 1 diabetes whom are associated with low bone mass and early onset of bone diseases (84).

**Table 1:** Key cellular components, their origins, targets, and physiological effects.

Signalling molecule/factor/gene	Origin/role	Targets	Physiological effect	References
Esp	Osteoblast	PTP	Regulate osteocalcin by acting on insulin pathway in osteoblasts	(103)
HIF-1	Transcription factor that responds to hypoxia	Specific DNA sequences, binds to VHL, VEGF	Increases glycolysis, promotes angiogenesis, supports tumour survival under hypoxic conditions	(90, 104)
Leptin	Peptide hormone	Hypothalamus	Inhibits bone formation centrally, peripherally, it promotes osteoblast activity	(105, 106)
Osteocalcin	Osteoblast	Pancreas	Insulin secretion, enhances energy expenditure, and supports bone formation	(107, 108)
Osteoglycin	Myoblastic cell	Y1 receptor	Supress bone formation by reducing osteoblastic activity	(109)
von Hipple Lindau	Tumour suppressor gene	HIF-1α	Regulates degradation of HIF-1α under normoxic conditions, affects tumour suppression by inhibiting HIF-1α-mediated pathways	(88, 110)
Wnt	Secreted glycoprotein that mediate cell-cell communications	β-Catenin, Lrp5	Induces aerobic glycolysis and regulates osteoblast maturation, involved in tumour growth signalling	(97, 111)

When mammalian cells are exposed to hypoxia in tissue culture, they increase glycolysis, a response thought to be cell-intrinsic. This is driven by the post-translational stabilisation of the hypoxia-inducible factor HIF-1 (85, 86). Under normal oxygen conditions, HIF-1 is continuously produced, but its levels are regulated post-translationally. Prolyl hydroxylase enzymes hydroxylate specific proline residues on the HIF-1α subunit, leading to recognition by the von Hippel-Lindau tumour suppressor (pVHL) and subsequent ubiquitination, targeting HIF-1α for degradation (87, 88). A study on the role of bones in glucose metabolism found that excessive glycolysis in osteoblast lineage cells that is induced by hypoxia signalling increases the use of glucose within the skeleton, thus reducing systemic levels of glucose (89). A pre-clinical study in mice showed that mice with the induced deletion of the hypoxia signalling pathway component von Hippel-Lindau (VHL) in skeletal osteolineage cells led to high bone mass of mice but also hypoglycaemia and increased glucose tolerance not accounted for by osteocalcin or insulin. *In vitro* and *in vivo* data indicated that VHL deficient osteoblasts had increased glucose uptake and glycolysis associated with hypoxia inducible factor (HIF) - target gene expression, resembling the Warburg effect that affects cancer cells (90). The improved metabolism in VHL mutant mice (90) is associated with increased tumour growth and metastasis (91). HIF signalling activation also increases bone mass likely through multiple key downstream effecters including vascular endothelial growth factor (VEGF) that mediates coupled osteo-angiogenic responses in bone. Local HIF activation in bone cells triggers increased glycolysis, which is associated with strongly enhanced osteoblastic glucose consumption (92). Studies with human patients with bone metastases from adenocarcinoma lung cancer (stage IV) showed that the level of glucose uptake in metastatic lesions inversely correlated with blood glucose levels and glycated haemoglobin values (HBa1C) (90). Overall, literature published supports a link between osteoblast-secreted osteocalcin and systemic glucose regulation (**Figure 3**).

Wnt pathways are a group of signal transduction pathways that send signals from proteins through cell surface receptors. The processes that it controls include body axis patterning, cell fate specification, cell proliferation and cell migration in bone, heart and muscle (93). Moreover, Wnt impacts bone homeostasis through β-catenin dependent and independent pathways. Activation of Wnt/β-catenin stimulates expression of transcription factor, osterix, promoting osteoblast differentiation (94). A study on glycogen synthase kinase 3β, a negative regulator of Wnt, showed that its deletion decreased circulating glucose secondary to increased insulin sensitivity. The blood glucose levels of these mutant mice had switched from low to high suggesting insulin resistance which is what occurs in feature Type 2 diabetes (95). This was supported by a study conducted by Yao et al. who showed that whole-body metabolism is regulated by osteocalcin and, specifically, through Wnt/β-catenin signalling pathway in osteoblasts (96). Moreover, another study by Kim et al. concluded that Wnt signalling through low-density lipoprotein-related receptor 5 (Lrp5) coreceptor regulates osteoblast maturation and whole-body glucose metabolism. Mice lacking Lrp5 in osteoblasts had hyperglycaemia, reduced glucose tolerance and insulin sensitivity reinforcing a link between the Wnt signalling pathway and osteoblast insulin signalling suggesting it could influence whole-body glucose homeostasis (97). A pre-clinical study also highlighted the role of delta like-1 (DLK1) in bone, showing that uncarboxylated osteocalcin controls production of DLK1 which works as a negative feedback system preventing osteocalcin induced hypoglycaemia (98). These studies provide further evidence of the role of bone cells in regulating whole-body glucose levels.

Osteoglycin, coded by the OGN gene and a secreted proteoglycan is expressed in a variety of organs and has an impact on bone formation, tumourigenesis amongst many other roles (99). Osteoglycin inhibits osteoblast activity whilst feeding back to increase whole-body homeostasis acting centrally via neuropeptide Y (NPY) neurons. Periods of low osteoglycin, seen in obesity, reduce food intake and insulin sensitivity, thus increasing serum glucose availability. A study showed that NPY signalling directly through peripheral Y1 receptors within osteoblasts controls insulin secretion and regulates glucose homeostasis (100). Another study shows that this central NPY signalling initiated process leads to subsequent osteoblastic Y1 signalling increasing osteoglycin secretion when energy balance is low (101). This study also uses an osteoglycin knockout mice model showed that osteoglycin acts to supress bone formation by supressing osteoblastic activity. It also feeds back to help increase whole body energy balance by acting centrally on hypothalamic NPY-ergic neurons to increase food intake as well as by improving glucose uptake through modulation of insulin secretion and insulin action at target tissue such as muscle. Conversely, the low levels of circulating osteoglycin seen during obesity act dually to both reduce food intake and reduce insulin responsiveness, glucose uptake, and, as a consequence, increase blood glucose. The shift in blood glucose in turn increases the energy available to bone formation through increased glucose uptake into early osteoblasts, further enabling the skeleton to adapt to increases in body weight. The study also showed that in humans post gastric surgery, as a model of negative energy balance, osteoglycin is associated with lower body mass index (BMI) and lean mass as well as changes in weight and glucose levels. This highlights the importance of Y1 receptor and osteoglycin in bone in the regulation of glucose homeostasis. Moreover, it identifies a role for osteoglycin in facilitating the matching of bone growth to alterations in energy status. This is further shown in a commentary of a study on osteoglycin, linking bone and energy homeostasis (102). In the study, researchers induced obesity in mice with a high fat diet. The authors reported that obese mice had decreased circulating levels of osteoglcyin compared with control mice fed normal chow. This effect was negatively associated with bone mass, which suggests that osteoglycin promotes alterations in bone mass following changes in body weight during intervals of high-fat feeding. Moreover, the role of osteoglycin was further demonstrated in 22 patients who had underwent weight loss through dietary programme or gastric surgery (102). Before weight loss, there was a negative correlation between osteoglycin levels and BMI, and a positive correlation between osteoglycin levels and lean mass. After the intervention, patients who had undergone substantial weight loss had a considerable increase in levels of osteoglycin, which correlated with reduced blood levels of glucose. Therefore, osteoglycin in humans is linked with regulating glucose metabolism during changes in energy homeostasis, which supports the findings in mice (102).

Overall, the mechanistic and clinical studies outlined above had clear outcomes confirming the role of bones in regulating whole-body glucose metabolism through a variety of pathways. These studies are beneficial since they untangle complex disease mechanisms and propose new avenues for research into understanding the initiation and progression of human systemic disease, and emerging therapeutic avenues that consider the skeletal system as an important player in the regulation of systemic glucose metabolism.

## Known and emerging links between cancer, systemic glucose, and bones as endocrine tissues

Malignant cells support tumour growth by adapting to metabolic changes. The link between whole-body glucose metabolism and tumourigenesis has attracted great interest from researchers and clinicians. The following studies discuss the emerging research on the links between cancer, systemic glucose and bones as endocrine tissues.

Previously published studies showed that osteoprogenitor cells (OPCs) are found in hypoxic environments in the bone marrow and that activation of HIF signalling in these cells increases bone mass, but also favours breast cancer metastasis to local bones (91). HIF signalling in osteoblast lineage cells also promotes breast cancer growth and dissemination remotely, in lungs and in other distant tissues from bones (91). These results indicate that that loss of bone homeostasis through alterations of the bone anabolism could affect breast cancer progression and present the skeleton as an important organ of the tumour macroenvironment. They also suggest that targeting the bone microenvironment could limit systemic tumour growth and dissemination in breast cancer.

**Figure 4:**
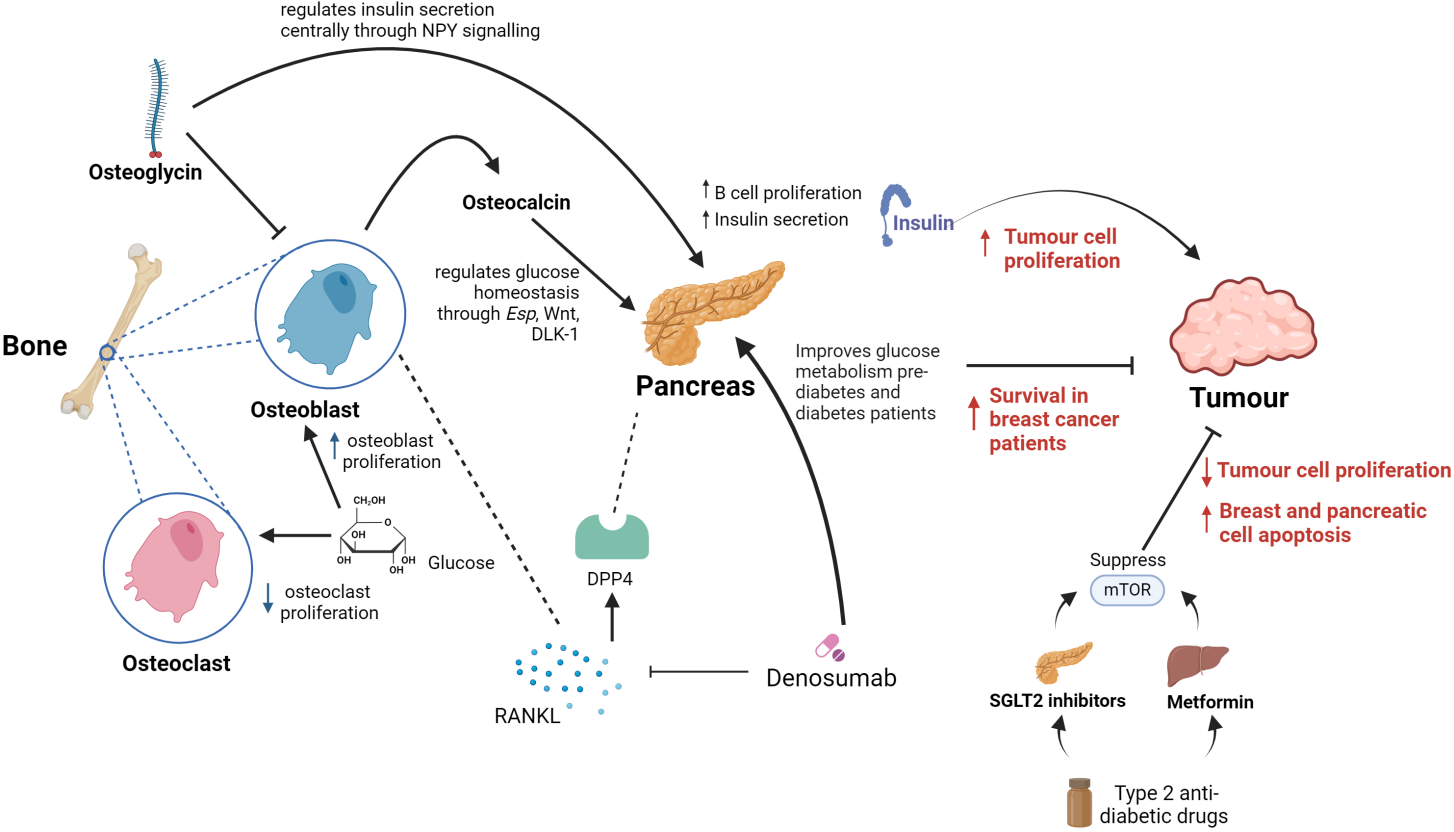
Summary of proposed key links between bones, systemic glucose and cancer. In bone, osteoclasts and osetoclasts are affected by glucose metabolism. Glucose causes reduced osteoclast proliferation and increased osteoblast proliferation. Osteoblasts in bone release the hormone osteocalcin, which is responsible for multiple endocrine functions including acting on the pancreas to increase insulin secretion and beta cell proliferation. Osteoglycin inhibits osteoblast activity and regulates glucose homeostasis through NPY. Denosumab inhibits the RANKL receptor which is found in osteoclasts in bone and leads to increased survival in breast cancer patients. It also improves glucose metabolism in pre-diabetes and diabetes patients. DPP4 is a well-known enzyme involved in glucose homeostasis, including via pancreatic action, that is known to be an osteoclast-derived protein. Therefore, DPP4 is an important link between RANKL and systemic glucose metabolism homeostasis. Metformin and SGLT2 inhibitors used in the treatment of diabetes supress mTOR which has shown to reduce tumour cell proliferation and increase breast and pancreatic cell apoptosis.

Osteoglycin, as previously mentioned, is a protein that takes part in the development of the bone extracellular matrix and influences many processes including tumourigenesis (112). Several studies have shown that osteoglycin plays an important role in creating the cancer microenvironment and that it is in fact regulated by the p53, a tumour suppressor gene (113). Moreover, its role in controlling tumour cell proliferation by either increasing or inhibiting cancer growth exhibiting both protumourigenic and antitumourigenic properties have been identified in different cancers, including colorectal, breast and ovarian cancers (114, 115). Particularly in colorectal cancer, osteoglycin has been involved in enhancing the T lymphocyte infiltration (116). These data indicates that osteoglycin could be involved in regulating the tumour microenvironment and may be a useful therapeutic target in oncology. However, there is limited evidence on osteogylcin’s role in other tumours and in glucose homeostasis, further research on how bone-mediated systemic glucose homeostasis can impact cancer development and progression is needed (116). Notwithstanding, an *in vivo* study conducted in mice highlighted that short-term starvation, therefore glucose restriction, enhances chemotherapy drugs efficacy, promotes tumour deceleration and increases cancer-free survival in melanoma, glioma and breast cancer. This was due to limited toxicity in normal cells, reinforcing the utility of targeting glucose pathways for cancer therapies (117). To this regard, modulating systemic glucose via sodium-glucose co-transporter 2 (SGLT-2) inhibitor drugs, which target the mammalian target of rapamycin (mTOR) signalling pathway, reduced tumour cell proliferation and induction of breast cell apoptosis (118). In addition, Canagliflozin (an SGLT-2 inhibitor), also showed antiproliferative effects and induction of apoptosis through the mTOR signalling pathway in pancreatic cancers (119) (**Figure 4**).

Classic drugs that target critical metabolic control points for aerobic glycolysis may therefore act as an opportunity in treating cancer (**Figure 4**). This is due to the fact that high levels of insulin in the bloodstream increase mitosis and tumour cell growth and proliferation (120). There is a volume of research suggesting that the drugs that are used to treat metabolic diseases such as Type 2 diabetes could be utilised for cancer therapy. A retrospective clinical study has found that metformin, a widespread anti-diabetic drug, may offer an element of cancer prevention as well as better outcomes when used in combination with other cancer therapies (121). Potential anticancer properties of metformin are thought to be based on two different mechanisms. First, insulin is a growth factor and exerts mitogenic properties mediated through insulin-like growth factor receptors and insulin receptors. Metformin decreases insulin resistance and lowers circulating insulin levels, leading to decreased signalling on insulin-like growth factor receptors and insulin receptors (122). Metformin functions by targeting the enzyme activated protein kinase (AMPK) which encourages muscles to take glucose up from the blood and inhibits transcription of the gene responsible for glycogenesis in liver cells. A recent study has eluded that the upstream regulator of AMPK is a protein kinase known as LKB1 which is a well-known tumour suppressor. Moreover, metformin inhibits mTOR activity through activation of LBK1 and in turn AMPK, ultimately reducing protein synthesis and angiogenesis (123). Furthermore, a separate study has demonstrated that using new antiglycolytic agents such as dharichloroacetate have been tested for monotherapy in solid glycolytic tumours such as hepatocellular carcinoma by inducing cellular apoptosis and decrease cancer cell proliferation, improving survival in animal models (124).

Studies have shown that the long term use of insulin, in particular in individuals with Type 2 diabetes, has an increase in cancer risk. A study has shown that long term use of insulin, is associated with increased risk of ovarian cancer (122). Moreover another study suggests that insulin therapy, especially with certain analogs like insulin glargine, is linked to an increased risk of breast cancer. Previous studies have suggested that breast epithelial cells exposed to insulin are at risk for transformation in a stepwise carcinogenesis process (125). More specifically, insulin has been shown to activate members of the insulin-like growth factor (IGF) receptor family to inhibit apoptosis and subsequently prolong the survival of these transformed breast tissue cells (125). This mechanism suggests that insulin not only facilitates glucose uptake but may also support a tumour-friendly microenvironment, thereby affecting whole-body glucose metabolism and contributing to cancer progression (126). This interplay between insulin, glucose metabolism, and cancer risk underscores the importance of monitoring insulin therapy and metabolic health in cancer-prone populations. Moreover, future research should focus on elucidating the mechanisms by which insulin and glucose metabolism influence cancer development and exploring the efficacy of metabolic-targeting therapies in reducing cancer risk and improving patient outcomes.

Previous data show that once tumour cells disseminate to the bone marrow, there is increased chance of relapse of cancer resulting in particularly a poor prognosis in early stage breast cancer (127). In breast cancer bone metastases, tumour cells secrete cytokines and growth factors causing stroma cells and osteoblasts to secrete RANK-ligand (RANKL). This leads to increased osteoclast differentiation which in turn stimulate tumour cell proliferation and tumour cell survival (128). Another study shows that breast and prostate cancer cells not only express RANKL but also upregulate RANKL expression by osteoblasts and bone marrow stromal cells (129, 130). Numerous experimental models of bone metastasis have shown that RANKL antagonists prevent tumour-associated osteolysis and significantly reduced skeletal tumour burden (131). Animal models that mimic advanced prostate, breast, or non-small cell lung cancer, representing both osteolytic and osteoblastic skeletal lesions, have demonstrated that the RANKL inhibitors, RANK-Fc or OPG-Fc,were effective in preventing or delaying of bone metastases and reducing progression of tumours in the skeleton (132). This was observed in a model of carcinogen and hormone-induced breast cancer, which demonstrated that RANKL inhibition with RANK-Fc significantly delayed mammary tumour formation in transgenic mice and almost completely blocked tumour formation in wild-type mice (133). Denosumab is a drug which is a human monoclonal antibody and is used in the treatment of osteoporosis and bone metastases (134). It inhibits RANKL, which is involved in the bone remodelling cycle, and has resulted in disease free survival in patients with breast cancer (135) (**Figure 4**). Previous studies have also shown that inhibiting RANKL with denosumab improved glucose metabolism parameters in pre-diabetic and diabetic patients (136).

In the RANKL pathway, OPG is an inhibitor of RANKL, which enhances osteoclasts apoptosis (137). High OPG levels are associated with impaired glucose tolerance (IGT) which could be as a result of insulin levels decreasing in patients with IGT and the similar roles between OPG and insulin in blocking osteoclastogenesis (138–140). Based on OPG’s function, Denosumab, could improve glucose entry into the muscle through increasing insulin sensitivity (141). Denosumab was also proven to decrease dipeptidilpeptidase-4 (DPP4) serum concentrations and increase glucagon-like peptide-1 (GLP-1) in subjects with IGT, demonstrating a crucial role in glucose and insulin metabolism (142). DPP4 is a well-known enzyme involved in glucose homeostasis that is known to be an osteoclast-derived protein (117). Therefore, DPP4 is an important link between RANKL and systemic glucose metabolism homeostasis.

However, it must be understood that RANKL participates in other tissues. Studies show that RANK/RANKL system plays an essential role in developmental maturation and functional maintenance of the immune system (143, 144). Studies of mice with heart failure have also shown a high and persistent expression of the RANK, RANKL, and OPG genes in the ischemic and nonischaemic areas of the heart (145). Furthermore, intravenous post-infarction anti-RANKL treatments in mice have shown to reduce infarct size and cardiac neutrophil infiltration (146). In addition, in muscles, high levels of RANKL expression generates poor glucose uptake, leading to alterations in muscle metabolism (141). In the liver RANKL induces insulin resistance by promoting inflammation, whereas in the pancreas RANKL produces a hyperglycaemic state by decreasing insulin production and augments glucagon production which could lead to β-cell dysfunction (141, 142). The main sources of RANKL are macrophages that have infiltrated the bone, pancreas, liver and muscles, which is a signal for the inflammatory NF-κB pathway, linking it to impaired IGT (147–149). Studies have shown that metformin decreased RANKL activity and its expression in the bone and liver, however the molecular mechanisms are not fully understood (150, 151).

Drug clinical trials have shown that systemic glucose homeostasis can be regulated both by using classic glucose metabolism modulation (e.g. anti-diabetic drugs) or by using emerging glucose metabolic modulation by bone signalling (e.g. RANKL). Both have demonstrated impact on cancer progression, patients’ survival and disease outcomes. This adds plausibility to the hypothesis that bone metabolism could influence systemic glucose homeostasis with potential consequences for cancer progression and treatment.

## Challenges, conclusions and future perspectives

Glucose metabolism plays a role in cancer development and progression through many different mechanisms. Moreover, bone metabolism affects whole-body glucose homeostasis and in turn could be hypothesised that it can take part in cancer progression and treatment. However, further studies focussing on addressing the hypothesis of how bones impact glucose systemically, and how this changes the disease course in oncology need to be carried out. Understanding these dynamics is critical for developing integrated therapies that target both bone health and metabolic alterations in cancer patients. Furthermore, modulating pathways that influence bone remodelling may provide new avenues for controlling cancer metastasis to bone. For example, drugs that target energy metabolism, like metformin used in patients with diabetes, impact osteoblast or osteoclast activity, and in turn this can affect cancer progression. Moreover, inhibitors and activators of osteoblast differentiation and bone formation for example Wnt inhibitors, Esp and osteocalcin can alter bone formation and in turn, energy metabolism. Additionally, inhibitors of GLUT transporters could also be a target for modulating glucose metabolism, and in turn be used for anticancer therapies. Understanding the additional role of these drugs is important since this knowledge could ultimately be used to develop treatments to slow cancer cell growth and cause tumour death.

The exploitation of potentially reprogrammed glucose metabolism to target cancer cells either by classically targeting systemic glucose metabolism or bone glucose metabolism provides new worthwhile therapeutic targets and avenues. Future treatment of cancer patients should consider a more holistic approach where bone health and systemic glucose metabolism are important factors to be appraised and, when possible, modulated alongside conventional cancer therapy for specific tumour types (e.g. radiotherapy, chemotherapy, and targeted immunotherapy). Advances in understanding interactions between osteoblasts, osteoclasts, and bone metastatic cancer cells will aid in controlling and ultimately preventing cancer cell metastasis to bone.

## Data Availability

The original contributions presented in the study are included in the article/supplementary material. Further inquiries can be directed to the corresponding author.
